# Nitrous oxide: a unique official French addictovigilance national survey

**DOI:** 10.3389/fpubh.2023.1167746

**Published:** 2023-05-03

**Authors:** Marylène Guerlais, Aurélie Aquizerate, Arthur Lionnet, Amélie Daveluy, Mélanie Duval, Marie Gérardin, Marion Istvan, Edouard-Jules Laforgue, Caroline Victorri-Vigneau, for French Addictovigilance Network

**Affiliations:** ^1^Nantes Université, CHU Nantes, Service de Pharmacologie Clinique – Centre d’Evaluation et d’Information sur la Pharmacodépendance-Addictovigilance, Nantes, France; ^2^Nantes Université, CHU Nantes, Service de Neurologie, Nantes, France; ^3^Inserm, U1235, Nantes, France; ^4^Centre d’Addictovigilance, Département de Pharmacologie Médicale, INSERM, BPH, U1219, CHU de Bordeaux, Bordeaux, France; ^5^Nantes Université, CHU Nantes, INSERM, methodS in Patient-centered outcomes and HEalth ResEarch, Nantes, France

**Keywords:** nitrous oxide, substance use disorder, addictovigilance, pharmacodependence, addiction

## Abstract

**Introduction:**

Nitrous oxide has become over the last few years a public health problem in many countries. France has a dedicated health monitoring system dedicated to the surveillance of the abuse, dependence and consequences associated with the use of psychoactive substances coordinated by the French National Agency for the Safety of Medicines and Health Products.

We present the French national survey of nitrous oxide.

**Materials and methods:**

We analyzed all the cases with nitrous oxide from 2012 to 2021: number of notifications, characteristics of the subjects and consumption, consequences reported and their evolutions over time. In addition, we have made a special focus on the four main complications reported.

**Results:**

A total of 525 cases were received with an exponential increase since 2019. We observed changes in the characteristics of the notifications with an increase in the proportion of women [42.7% in 2021 vs. 30.8% in 2020 (*p* = 0.02)]; an increase in the quantities consumed (use of cylinders); a negative evolution of the contexts of use with a search for self-therapeutic effects and use in violent contexts; an increasing trend of the severity of cases [78.1% in 2021 vs. 70.0% in 2020 (*p* = 0.07)].

The main effects were substance use disorders and/or associated criteria (82.5%), neurological disorders (75.4%), psychiatric symptoms (15.4%) and cardiovascular events (8.6%). In terms of evolution, we observed a significant increase in cases with a use disorder and an increase in neurological complications. Moreover, new serious effects, notably cardiovascular events were reported.

**Discussion:**

The combination of high availability, varied effects from euphoria to relief of discomfort in a stressful global pandemic context and the development of dependence could explain the rapid growth of consumption and the seriousness of the cases.

It must now be taken into account that (i) Substance use disorders are associated with nitrous oxide consumption; (ii) clinicians must consider “nitrous oxide” in young subjects presenting different types of manifestations; and (iii) stopping consumption is imperative and is the first treatment. In this context, an addictological assessment must also be carried out.

## Introduction

### Nitrous oxide: a public health problem in many countries

Nitrous oxide can be used as a medicine available in prescription drugs or as a food additive that is freely accessible. In France, as in many other countries, nonmedical nitrous oxide has rapidly become established on the drug scene over the last 5 years. There are few data on the prevalence of consumption. At the international level, the Global Drug Survey (GDS) data from 35 countries around the world show a usage lifetime prevalence estimated at 23.5% in 2019, becoming the 13th most consumed substance among the volunteer respondents of the online survey, with variations between countries, reaching 38.6% in 2014 in the UK (1, 2). In France, at the national level, the increasing consumption of nitrous oxide is mentioned in the French Monitoring Centre for Drugs and drug Addiction (OFDT in French Observatoire Français des Drogues et des Tendances addictives) report “drugs and addictions, essential data” of 2019 (3) and in the report Emerging Trends and New drug (TREND in French Tendances Récentes et Nouvelles Drogues) of 2019 and 2020 (4). In England and Wales, in a sample of adults aged 16–59, the last-year consumption prevalence was found to be 2.3% in 2019 and reached 8.7% in individuals aged 16–24, positioning nitrous oxide as the second most used substance after cannabis in the latter population (5). Studies conducted in France found a high prevalence of consumption in student populations (6). These data make it possible to highlight the extent of consumption but do not make it possible to assess the consequences associated with consumption. Literature case reports allow the description of some complications but do not allow them to be classified according to their frequency.

### In France, a dedicated system globally for assessing psychoactive substances: the addictovigilance network

France is one of the only European countries with a dedicated surveillance system for cases of abuse and dependence on psychoactive substances. This surveillance relies on a network of 13 Centres for Evaluation and Information on Pharmacodependence—Addictovigilance (CEIP-A) (7) coordinated by the French National Agency for the Safety of Medicines and Health Products (ANSM). The organization and missions of these centers are defined in the Public Health Code (8). The main mission of this vigilance is to identify substances that give rise to abuse or dependence, to assess the risk and consequences in terms of public health and to provide information on substances and risks. This system complements the other monitoring systems (*i*) pharmacovigilance, which assesses the adverse effects of medicines and (*ii*) Toxicovigilance which monitors effects in humans following exposure to consumer products, plants, fungi or animals, based on data from the network of Poison Control Centres (PCs) from the telephone response to the emergency provided by the PCs.

Addictovigilance network operates through the notification by health professionals of cases of addiction, abuse and/or consequences related to the use of psychoactive substances. Indeed, in France, the notification of cases of abuse or dependence to the addictovigilance centers is a regulatory obligation for health professionals (9). Physicians, in accordance with the regulation, make clinical notifications of situations encountered in the context of their professional practice, including the assessment of use disorders and the diagnosis of the somatic or psychiatric consequences identified. They will logically make notifications for situations that seem unusual and/or serious to them. Thus the addictovigilance system fulfils an alert mission, and the number of notifications reported is not a reflection of consumption in our country but rather of the seriousness and/or unusual nature of the situations encountered by physicians, whatever their practice setting, hospital or private practice, specialist or general practitioner. Therefore, the evolution of the notifications, both quantitatively and qualitatively, is crucial in the analysis of a given substance, because it indicates the evolution of the number of serious situations and the evolution of the type of consequences reported.

In order to centralize the data analysis, a designated center is responsible for the national monitoring of a given substance. To assess the risks and the abuse/addiction potential associated with the use of a specific substance, all cases reported in France involving this substance are analyzed by the center responsible for the monitoring of this substance. This analysis allowed us to identify complications associated with substance use reported by health professionals at the national level.

The Nantes Addictovigilance Centre is responsible for the monitoring of nitrous oxide in France. In this work, we present the quantitative and qualitative analysis of all cases, including nitrous oxide, for which notifications were sent to the French addictovigilance network and their evolution over time.

## Methods

### Addictovigilance notifications

As previously mentioned, notifications are reported by health professionals. Notifications reach the centers directly using the official collection form (available on the ANSM website) or by using a notification portal set up by the health authorities.

An addictovigilance notification includes sociodemographic data (gender, age), data on the products consumed and the consumption (quantity, duration, search, and felt effects). Items from the Diagnostic and Statistical Manual of Mental Disorders (DSM) definition of substance use disorders are reported (tolerance, withdrawal, substance intake in larger amounts or over a longer period than intended, unsuccessful efforts to cut down or control substance use, great deal of time spent in activities necessary to obtain use or recover from substance effects, reduction of important social, occupational, or recreational activities because of substance use, continuation despite knowledge of having a persistent physical or psychological problem that is likely to have been caused or exacerbated by the substance and craving) (10, 11). Moreover, the psychiatric and somatic consequences of the consumption are detailed.

The notifications are analyzed by medical pharmacologists working in the addictovigilance centers. The analysis focuses on the severity of reported use disorders for each substance and on the consequences associated with use. The severity of the case is assessed using pharmacovigilance and addictovigilance severity criteria: hospitalization/extended hospitalization; life-threatening, death, disability/incapacity, congenital anomaly or other serious medical situation.

Then, reports are recorded in the French national database and then the European national database, respecting the regulatory deadlines for severe cases (13 and 15 days after receipt, respectively).

### Nitrous oxide survey

For this work, we selected all cases concerning actual voluntary consumption of nitrous oxide in the French national database, without any time limitations.

In the severity classification, we considered in “other serious medical situation,” any situation of daily and/or high dose use (≥ 20 cartridges or cylinders equivalent per occasion or day) as justifying a classification as a serious case (mentioned as substance use disorder-associated criteria).

For the analysis of psychiatric and neurological injuries, we asked for diagnosis classification by specialists in the field, psychiatrist (EJL) and neurologist (AL), respectively. Psychiatric symptoms were classified as anxiety, psychotic, cognitive, behavioral, affective and vigilance disorders. Neurologic injuries were classified as spinal cord syndromes (combined sclerosis, myelopathies), neuropathies, neurological symptoms and potentially neurological aspecific symptoms.

### Analysis

We provide information on the number of notifications, the characteristics of the subjects and consumption, and the consequences reported, with a special focus on the four severe most frequently reported consequences (use disorders, neurological, psychiatric, and cardiovascular consequences). For quantitative and qualitative analysis of notifications characteristics, we used continuous data expressed as the mean [± standard deviation (SD)] and categorical data as numbers and percentages (calculated on the total of the filled data).

We studied the evolution over time of the number of notifications in addition to the characteristics of the subjects and the reported consequences. Comparisons are used to assess evolution over time between 2020 and 2021 using standard tests, Chi2 for proportions and t tests for means.

## Results

### Number of notifications and evolution according to time

**Figure 1:**
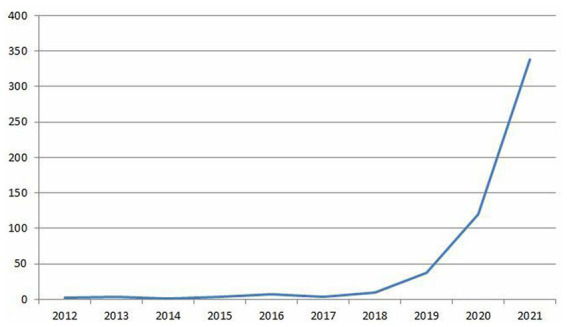
Number of nitrous oxide notifications reported to the addictovigilance network by year.

In total, 525 notifications of effects linked to the consumption of nitrous oxide were received by the French addictovigilance network. The first reports of nitrous oxide misuse (the misuse of cartridges by young people) were received in 2012. The number of notifications increased exponentially from 2018 to 338 notifications in 2021 (Figure 1 and Table 1). In our results, we compared the years 2021 with 2020, as the previous years correspond to the beginning of the exponential increase in the number of notifications (Figure 1). In absolute value, this number has increased almost tenfold between 2019 and 2021 (37 vs. 338), and the proportion has also increased to reach more than 6% of all notifications in the French addictovigilance network by 2021 (*p* < 0.001) (Table 1).

**Table 1:** Patient characteristics.

	Total 2012–2021	2020	2021	Comparisons 2020/2021
Nitrous oxide (NO) notifications	525	120	338	
Percentage of NO notifications		1.6% (120/7275^1^)	6.6% (338/5159^2^)	*p* < 0.001
Patients characteristics for NO notifications				
Sex (*men*)	60.7% (*n* = 318/524)*	69.2% (*n* = 83/120)*	57.3% (*n* = 193/337)*	*p* = 0.02
Age				
*Mean*	21.9 years (SD 4.7)*	22.2 years (SD = 5.2)*	21.6 years (SD = 4.4)*	*p* = 0.23
*Median*	21 years (13–53)*	21 years (21–53)*	21 years (13–41)*	
*< 18 years*	11.3% (58/515)*	12.6% (*n* = 15/119)*	11.2% (*n* = 37/330)*	*p* = 0.68
NO consumption characteristics				
Forms and dose used				
*Cylinders*	54,6% (*n* = 191/350)*	31.8% (*n* = 28/88)*	73.0% (*n* = 162/222)*	*p* < 0.001
Frequency of use daily or more	48,5% (*n* = 157/324)*	36.0% (*n* = 27/75)*	52.4% (*n* = 110/210)*	*p* = 0.01
Anteriority of use (*mean in months*)	16.5 months (SD 16.7)*	19.3 months (SD = 18.3)*	15.5 months (SD = 16.2)*	*p* = 0.17
Associated consumption				
*At least one, other than tobacco*	36.4% (*n* = 191/525)	40.8% (*n* = 49/120)	36.7% (*n* = 124/338)	*p* = 0.42
*Alcohol*	23.4% (*n* = 123/525)	20.8% (*n* = 25/120)	22.8% (*n* = 77/338)	
*Cannabis*	23.8% (*n* = 125/525)	27.5% (*n* = 33/120)	23.1% (*n* = 78/338)	
*Other*	12.8% (*n* = 67/525)	14.2% (*n* = 17/120)	10.9% (*n* = 37/338)	
Presence of severity criteria	74.5% (*n* = 391/525)	70.0% (*n* = 84/120)	78.1% (*n* = 264/338)	*p* = 0.07
Effects sought	Euphoria, anxiolytic, relaxation/well-being, “high”/“stoned” or amnesia	Emergence of uses for self-treatment effects with daily consumption and uses in violent context of aggression or prostitution		

The “*” means “missing data”.

### Characteristics of the notifications and evolution according to time

A description of the patients and use characteristics is presented in Table 1 together with the evolution over time.

Men account for the majority of cases in the notifications, however, we observed an increase in the proportion of women (42.7% in 2021 vs. 30.8% in 2020 (*p* = 0.02)).

Concerning the form used, cartridges and cylinders of various sizes (including “tank” or “obus”) are reported, sometimes several forms per subject. There is little precision on the quantity contained in the containers which seem to vary in volume from 75 mL to 5 L and in weight from 580 g to 4 kg. The most commonly mentioned containers appear to be cylinders of about 600 g. The quantities consumed range from 1 cartridge to 48 cylinders per occasion and from 1 cartridge to 2 “tanks” or 24 cylinders per day. In 2021, the consumption of one or more cylinders per day or per week was reported in 35.9% of cases (*n* = 84/234). In 2021, the notion of using cylinders is reported in 73% of the notifications vs. 31.8% in 2020 (*p* < 0.001) and daily or more consumption increased from 36% in 2020 to 52.4% in 2021 (Table 1).

Concerning searched effects contexts of use, we have gone from a festive use with a search for euphoria to a search today for self-therapeutic effects as amnesia or better being; moreover, we observed a negative evolution of the contexts of use with a use in violent contexts like prostitution or before aggression.

The vast majority of cases were classified as “severe.” But we note an increasing trend of the severity of cases [78.1% in 2021 vs. 70.0% in 2020 (*p* = 0.07)].

Moreover, a major impact of consumption on care was reported in 2021: refusal of treatment, discharge against medical advice, noncompliance in 43 notes (12.7%), the recurrence of several hospitalizations for different effects in 12 notifications and a new phenomenon, the self-administration of vitamin B12 for preventive purposes.

Various consequences have been reported; sometimes the same subject presented more than one effect. The main effects, substance use disorders and/or associated criteria (82.5%), neurological disorders (75.4%), psychiatric symptoms (15.4%) and cardiovascular events (8.6%), are described in Table 2. For the “others” category, the following are reported: digestive effects (nausea, vomiting, abdominal/epigastric pain, transit disorders … *n* = 58, 11.0%), asthenia (*n* = 35, 6.7%); cold burns mainly on the hands, thighs (use of cylinders) and orobuccal area (*n* = 16, 3.0%); and falls causing potential trauma, including head injuries with coma (*n* = 11, 2.1%). Road traffic accidents related to nitrous oxide use were reported in 11 notifications (2.1%). The vast majority of accident notifications mention driving under the influence of nitrous oxide. Nitrous oxide was directly implicated in one death, following massive pulmonary oedema after inhalation of the gas, in a second death indirectly (in a road accident in which the passenger of the car whose driver was under the influence of nitrous oxide died) and is one of the substances described in the deaths of two other poly-consumers. Regarding the evolution of reported effects, there is an increase in cases with a use disorder (89.3% in 2021 vs. 73.3% in 2020; *p* < 0.001) and an increase in neurological complications (79.9% in 2021 vs. 69.2% in 2020; *p* = 0.02). Moreover, new serious effects, notably cardiovascular events were reported.

**Table 2:** Reported consequences.

Consequences (*Potentially more than one per subject*)	2012–2021 *N* = 525 notifications	2020 *N* = 120 notifications	2021 *N* = 338 notifications	Comparisons 2020/2021
Substance use disorders (SUD) and/or associated criteria (daily use and/or dose ≥20 cartridges/occasion or cylinders equivalent)	82.5% (*n* = 433) · Use disorders, abuse, drug dependence or addiction (with or without associated criteria) 78.3% (339/433) · Only associated criteria 21.7% (94/433)	73.3% (*n* = 88)	89.3% (*n* = 302)	*p* < 0.001
58.0% (51/88)42.0% (37/88)	85.8% (259/302)14.2% (43/302)	*p* < 0.001		
Neurological complications	75.4% (*n* = 396) · Myelopathy 59/396 (14.9%) · Neuropathy 41/396 (10.3%) · Myelopathy and neuropathy 47/396 (11.9%) · Neurological symptoms only 136/396 (34.3%) · Aspecific symptoms only 69/396 (17.4%) · Neurological and aspecific symptoms 44/396 (11.1%)	69.2% (*n* = 83)	79.9% (*n* = 270)	*p* = 0.02
Psychiatric symptoms (*Potentially more than one per subject*)	15.4% (*n* = 81) · Behavioral 36/81 (44.4%) · Psychotic 34/81 (42.0%) · Anxious 29/81 (35.8%) · Affective 19/81 (23.5%) · Cognitive 19/81 (23.5%) · Vigilance disorders 15/81 (18.5%)	24.2% (*n* = 29)	11.2% (*n* = 38)	*p* < 0.001
Digestive disorders (mainly nausea vomiting)	11% (*n* = 58)	11.7% (*n* = 14)	10.4% (*n* = 35)	*p* = 0.69
Cardiovascular events	8.6% (*n* = 45)	5.8% (*n* = 7)	7.7% (*n* = 26) Novel effects including thrombotic effects associated with hyperhomocysteinemia and risk factors in 2021	*p* = 0.50
Others	16.8% (*n* = 88)	29.2% (*n* = 35)	12.4% (*n* = 42)	*p* < 0.001

### Focus on substance use disorders (SUD)

Of the 433 notifications reporting a use disorder or associated criteria, in 94 cases, the only criterion reported was daily consumption and/or high dose (more than 20 cartridges/occasion or equivalent in a cylinder) (Table 2). The 339 other notifications were classified as substance use disorders. In 111 cases, DSM substance use disorder items were evaluated. All items of the DSM definition of substance use disorders are described through the reported cases: tolerance (*n* = 31), signs of withdrawal including hospitalization if necessary (*n* = 13), dose consumed or length of consumption (*n* = 67), desire to stop or previous unsuccessful attempts (*n* = 34), time spent obtaining/consuming/recovering from the effects (*n* = 45), harmful to social/relational aspects, including family, employment, financial or legal impact (*n* = 26), or health consequences including persistence of consumption after initial hospital treatment for somatic consequences related to nitrous oxide consumption (*n* = 39). Craving is reported in 17 notifications. Risky behavior is reported in three cases (consumption while driving). The occurrence of addiction care or referral for addiction care is reported in 66 cases, the first time in 2017 and the other cases from 2020. In 51 cases (77.27%), the care was provided; in 15 other cases, the care was refused by the patient (or the patient ran away).

In terms of evolution, there was an increase in the severity of cases with a greater proportion of SUDs notified (Table 2).

### Focus on neurological complications

Among the 396 reports with neurological disorders that occurred after nitrous oxide consumption, the effects reported are divided into the following (see Table 2):

- Myelopathy, with or without neuropathy, in 26.8% of cases (*n* = 106), including subacute combined degeneration of the spinal cord (*n* = 74).

- Neuropathy or polyneuropathy, with or without myelopathy, in 22.2% of cases (*n* = 88).

In total, 47 cases combined the two types of disease (central and peripheral). In 28.6% of the cases of central and/or peripheral injuries (*n* = 42/147), an associated consumption (other than tobacco) was reported [cannabis (*n* = 28), alcohol (*n* = 23), cocaine (*n* = 6), benzodiazepine (*n* = 2), poppers (*n* = 1), or tramadol (*n* = 1)].

- Neurological effects, not sufficient for the diagnosis of myelopathy and/or neuropathy, in 62.8% of cases (*n* = 249), including the following: (i) neurological symptoms (*n* = 180, 45.4%), mainly paraesthesia, hypoesthesia, sensory-motor deficits, gait disorders and ataxia; and (ii) aspecific neurological symptoms (*n* = 113, 28.5%), mainly headache, malaise/vertigo and cognitive disorders. Loss of consciousness/coma was reported in 19 cases. In 32.1% of these cases of neurological effects (*n* = 80/249), an associated consumption (other than tobacco) was reported [alcohol (*n* = 48), cannabis (*n* = 44), cocaine (*n* = 13), ecstasy/MDMA (*n* = 6), poppers (*n* = 4), benzodiazepine (*n* = 3), codeine (*n* = 2), and pregabalin (*n* = 2); the other substances were reported once (methamphetamine, cyproheptadine, deodorant, heroine, ketamine, synthetic cannabinoid, neuroleptic)].

Overall, the proportion of cases with associated substances is 30.8% (*n* = 122/396) for notifications reporting neurological complications vs. 53.5% (*n* = 69/129) (*p* < 0.001) for other notifications.

Among severe neurological cases, 40.2% were women in 2021 (*n* = 90/224) vs. 24.2% in 2020 (*n* = 16/66) (*p* = 0.02).

In 160 cases, a vitamin B12 dosage was reported; a deficiency was reported in 91 cases (56.9%), a normal level with a low limit in 13 cases (9.1%) and a normal level but with prior supplementation in 5 cases (3.1%). A homocysteine assay was reported in 81 notifications, of which 78 cases (96.3%) with hyperhomocysteinemia were found. Methylmalonic acid dosage was reported in 27 cases, with elevated levels found in 21 cases (77.8%). Blood count data were available in 68 cases: anemia was reported in 17 cases (25.0%) [macrocytic (*n* = 6), are generative (*n* = 2)] and macrocytosis in five cases. Other haematologic abnormalities were reported: neutropenia in one case, and it should be noted that in another case, without neurological disorders mentioned, pancytopenia was reported.

Treatment with vitamin B12 supplementation (mainly IM) was mentioned in 149 reports, and rehabilitation was reported in 61 cases.

### Focus on psychiatric manifestations

Several types of psychiatric manifestations were reported (several possible types of manifestations per patient), mainly behavioral manifestations, psychotic manifestations and anxious manifestations (Table 2).

Of the 81 notifications in which clinical psychiatric signs were reported, an associated consumption was reported in 42 cases (51.85%), as opposed to 33.6% (*n* = 149/444) for other notifications (*p* < 0.001).

### Focus on cardiovascular events

Of the 44 notifications in which clinical cardiac symptoms were reported, these were mainly changes in heart rhythm clinically proven or felt by the subject [tachycardia (*n* = 11), bradycardia (*n* = 6)] palpitations (*n* = 7) and chest pain sometimes with radiation (*n* = 15). Left ventricular dysfunction (in the context of polyintoxication and coma) and a stroke with dissection of the left carotid artery without thrombosis have also been reported.

At the vascular level, in 2019, the first case of thrombotic origin was reported: a bilateral pulmonary embolism and unilateral deep vein thrombosis (with other risk factors). In 2021, 7 other notifications reported thrombotic effects in the context of hyperhomocysteinemia and other risk factors in subjects consuming one to several nitrous oxide cylinders per day or per occasion (when the information is provided): pulmonary embolism (*n* = 3); vein thrombosis (*n* = 4), including 2 cases of deep vein thrombosis with double involvement and one case of cerebral vein involvement; and a case of acute coronary syndrome with acute thrombotic occlusion of the right coronary artery.

## Discussion

Our work highlights an exponential increase in reports of complications related to nitrous oxide consumption in France, with a diversification and aggravation of the complications reported. These data represent the most important number of cases published to date. From our results, several points should be highlighted.

### Amplification of the phenomenon from 2018

The first reports in 2012 described the misuse of cartridges by young people and the modalities of this misuse; from 2018, together with the exponential increase in reports, notifications of serious consequences appeared. This led health authorities in France to conduct several communications (12–18). This increase in complications is taking place at the same time that an increase in consumption is observed in France and at the international level: the Global Drug Survey (GDS) data show a sharp increase in the prevalence of use over the last 12 months, from 6.3% in 2014 to 11.9% in 2019 (1, 19).

There are several factors that have contributed to the spread of the product and the increase in consumption (20–24): the low cost, the ease of obtaining it through the home deliveries that began with the COVID-19 lockdown in 2020 (24) and the parallel development of cylinders corresponding to several tens or hundreds of cartridges, sometimes with added flavors (22). These conditions are attractive for young individuals (20, 21, 25) in a period of brain development with vulnerability to risk-taking and health consequences (21, 26). This combination of high availability, varied effects from euphoria to relief of discomfort in a stressful global pandemic context and the development of dependence explains the rapid expansion of consumption and the seriousness of the cases (24). The misuse of nitrous oxide among the young population is now considered a public health problem in many countries (20, 25, 27). In France, the increase in consumption and the seriousness of the cases led to the promulgation of a law in June 2021 (28). This law now prohibits the sale or offer of nitrous oxide to any minor, the sale or offer of this product in bars, discotheques, student parties, etc., and tobacco shops. Moreover, the problem identified concerns the consumption of very large quantities of nitrous oxide. Therefore, a decree limiting the maximum quantity authorized for sale to individuals to the equivalent of 10 cartridges has been proposed. The impact of this regulation must be monitored in the future.

### Much more varied effects than previously thought

Even if its mechanism of action is not fully elucidated, nitrous oxide acts on the μ-opioid receptor (29–31) and on the noradrenergic, NMDA/glutamate and GABAergique systems (31–33). Furthermore, nitrous oxide induces (*i*) oxidation of the active center of cobalt contained in the chemical structure of vitamin B12 (34), leading to its inactivity and making it impossible for the transformation of methylmalonic acid and homocysteine (to methionine). Functional deficiency leads to an increase and a decrease in methionine necessary for the synthesis of myelin sheaths (20, 35, 36). Nitrous oxide also causes (*ii*) perturbation in the synthesis of nucleic acids, for which vitamin B12 is a cofactor, and is responsible for hematological anomalies (such as macrocytic anemia, leukopenia and even pancytopenia) (36). Finally, because of its mode of consumption, i.e., inhaled pure without oxygen, its recreational administration is generating hypoxia (20, 37). The central action explains the anxiolytic, analgesic and euphoric effects of the “laughing gas” demonstrated in the nineteenth century (38, 39), and the effects on vitamin B12 explain other consequences. Some of these are reported in the nitrous oxide mixed with oxygen (EMONO) approval used in medical settings (40), i.e., vitamin B12 deficiency, psychiatric conditions including pharmacodependence, paraesthesia and some neurological symptoms, dizziness, neurological disorders and myeloneuropathies after prolonged or repeated exposure. Indeed, EMONO approval refers to clinical use; however, whether in clinical or recreational use it is the same psychoactive substance. Neurological complications are well described (34, 37, 41, 42), as well as their main biological mechanism linked to vitamin B12 deficiency. These are the most frequently reported in the declarations of addictovigilance and are present in 3/4 of the cases, with no associated consumption in more than 70% of cases of myelopathy or neuropathy.

Use-related disorders and/or associated criteria are present in 433 notifications, i.e., in 8 cases out of 10.

These complications could have been expected, given the pharmacological properties (action on opiate receptors and GABA-A receptors). The nitrous oxide approval mentions “pharmacodependence,” although abuse and dependence are mentioned in a few published cases of somatic complications but without detailing the different items of abuse and dependence (43, 44). Only for a very few cases were DSM items such as tolerance and withdrawal reported (42, 45–47), and to our knowledge, only one published case fulfilled the *DSM-IV*-TR criteria of substance dependence (48). In a first article (49), we analyzed the nitrous oxide cases reported to the whole addictovigilance network without any time limit until 2016 and including the cases concerning EMONO. At that time, we found 21 cases and the cases reported mentioned mainly misuse; few DSM items were filled in. Today, we highlight a worrying evolution of nitrous oxide misuse and the occurrence, in addition to the known effects, of real substance use disorders. We find in the notifications (*i*) the description of the characteristic of positive conditioning at the beginning (consumption with anxiolytic aim, well-being to relieve a suffering), followed by negative conditioning (impossibility of stopping because of signs of withdrawal); and (*ii*) Use disorders were reported as such in the French national database in 339 cases. Moreover, in 111 cases items of DSM 5 were described in accordance with the data reported in the addictovigilance notifications (11) and all the criteria were reported in the different cases.

Although nitrous oxide has long been described as having a low addictive potential (20, 50, 51), our data indicate that it is a psychoactive substance with real dependence potential. Moreover, repeated and chronic consumption and high doses are associated with dependence and are therefore responsible for the various and serious somatic consequences. It is thus essential to manage the use disorders in patients using nitrous oxide, as we observed in our data proposals for the management of addictive care implemented mainly from 2020 onwards, and to set up protocols for health professionals that do not exist today.

Other complications not mentioned in the EMONO approval reported in recent years can be linked to pharmacological properties, especially cardiovascular complications. Hypoxia caused by inhalation of pure nitrous oxide without mixing with oxygen could explain the tachycardia, arrhythmia, and chest tightness sensation, which can lead to death by cardiac arrest (20, 37). Cardiovascular complications related to thrombosis for which hyperhomocysteinemia is a risk factor (52–55) have been reported more recently. The first case report was published in 2018 (56), and 14 case reports were published at the end of 2021 (PubMed database). The first French notification was reported in 2019, followed by 7 others life-threatening consequences (pulmonary, cardiac, etc.). These complications will need to be monitored, especially as our results show an increase in the proportion of women who are likely to be taking contraceptives and therefore to have more risk factors.

Hematological complications are rare but are found in French clinical cases and in some cases reported in the literature (57–59).

Psychiatric disorders are present, but fewer have been reported (37, 60, 61). The 81 French cases with reported psychiatric manifestations following nitrous oxide use may seem high in relation to these international data. We found a large diversity of the typology of psychiatric disorders observed in our results. The central mechanism of action of nitrous oxide may certainly be associated with some of these complications, including those listed in the approval. However, it is sometimes very difficult to link them to nitrous oxide because (*i*) the subjects sometimes have psychiatric comorbidities and (*ii*) this is the category of consequences where we found the most associations with other substances. However, even if the exact pathology underlying the psychiatric symptoms is unknown, some authors have hypothesized a mechanism for psychiatric toxicity as symptoms of vitamin B12 deficiency (60, 62); the increase in nitric oxide, antagonism of the N-methyl-D-aspartate (NMDA) receptor and induction of dopamine release in the nucleus accumbens could also play a role in the occurrence of disorders (60, 62). Health professionals should be aware of patients presenting with psychiatric symptoms (60) and include nitrous oxide in the drug history of patients who present with unexplained psychiatric abnormalities (62).

Finally, deaths are rarely reported in French addictovigilance notifications. Nevertheless, in 2016, Garakani et al. counted 11 publications corresponding to 29 cases reporting nitrous oxide-related deaths in which nitrous oxide was presumed to be the primary cause (37). The reported cause of death was acute asphyxiation due to hypoxia, sudden cardiac arrhythmia or sudden death (20, 37, 63). Due to the short half-life of nitrous oxide and rapid clearance through the lung, it is difficult/impossible to detect nitrous oxide in biological tests (20, 64, 65), and indirect deaths, including cases of road accidents following nitrous oxide inhalation, are regularly reported by the French media, but they rarely constitute addictovigilance notifications.

### Strengths and weakness

The main strength of this work is that it is based on data from an official surveillance network, addictovigilance, coordinated by the French health authorities. The notifications come from all over the country, are reported by health professionals and are thus rich in clinical elements, enabling an analysis of complications. Nevertheless, we analyze the data available to us and it must be kept in mind that the consumption data reported by the physicians are declarative data from the patients. We cannot exclude that some consumptions are not mentioned by the patients and that there is a variability in the transcription of these elements by the physicians. Moreover, in our medical addictovigilance data, there is a lack of demographic characteristics of the sample, like education level, income, ethnic minority. Such information would be highly valuable to identify subjects or subgroups at increased risk. Further studies incorporating these data would be required.

The number of addictovigilance reports, like any other monitoring system based on spontaneous reporting, is not a direct reflection of consumption in a country. Rather, these reports are a reflection of the severity of the consequences associated with a substance consumption. Of course, one can imagine that the more users there are, the more consequences will be reported. Nevertheless, one of the weaknesses of vigilance systems based on notifications by health professionals is underreporting, particularly of less severe consequences. Currently, digestive effects (nausea/vomiting, abdominal/epigastric pain…), for example, are far from being the most reported, although they are mentioned as “frequent” (meaning >1/100 to <1/10) in the EMONO approval (40). This is also the case for falls, which are rarely reported to the French addictovigilance network (11 times in 10 years), burns, and asthenia. This could be explained by the main mission of the French addictovigilance system, which is the alert. Cases are reported by health professionals. Professionals report complications in nitrous oxide users, causal relationship must be interpreted taking into account the pharmacological analysis and the presence of other risk factors. As a consequence, they concern subjects who have used the care system and thus are serious clinical cases. With regard to nitrous oxide, three-quarters of the reported cases were serious, and despite underreporting, their number is increasing exponentially.

## Conclusion—clinical implication

In the last decade, the recreational use of nitrous oxide has become a public health problem in several countries, including France. The French national follow-up data are unique and allow a hierarchization and characterization of severe clinical consequences. It must now be taken into account that (*i*) substance use disorders are associated with nitrous oxide consumption and (*ii*) the consequences of consumption are very varied and can be very severe. Thus, clinicians must investigate nitrous oxide use in young subjects presenting unexplained neurological signs, cardiovascular signs, psychiatric manifestations and possibly more minor symptoms such as falls, traumatisms (including traffic accidents) or even burns. Faced with a biological assessment, it is necessary to keep in mind that the vitamin B12 dosage may be normal, but an elevated homocysteine level is a marker of functional vitamin B12 deficiency (36). (*iii*) Stopping consumption is imperative and is the first treatment. In this context, an addictological assessment must also be carried out with an orientation if necessary.

## Data availability statement

The datasets presented in this article are not readily available because data from this study can be transmitted by the corresponding author upon reasonable request. Data source: National Agency for the Safety of Medicines and Health Products. The content of this article is the sole responsibility of the authors and has not been validated by the ANSM. Requests to access the datasets should be directed to CV-V, caroline.vigneau@chu-nantes.fr.

## Author contributions

MGu contributed to data collection, analysis, and the manuscript redaction. AA and MGe contributed to data collection. AL and E-JL confirmed the diagnosis in the field of psychiatry (E-JL) and neurology (AL). AD contributed to analysis. MD and MI contributed to analysis and the manuscript redaction. FAN contributed to the data collection. CV-V contributed to the analysis and wrote the manuscript. All authors read and approved the final manuscript.

## French Addictovigilance Network

Alexandra Boucher, Anne Batisse, Anne Roussin, Bernard Fauconneau, Cécile Chevallier, Frédéric Libert, Hélène Peyrière, Joëlle Micallef, Juliana Tournebize, Maryse Lapeyre-Mestre, Michel Spadari, Nathalie Fouilhé, Reynald Le Boisselier, Sylvie Deheul, Valérie Gibaja.

## Conflict of interest

The authors declare that the research was conducted in the absence of any commercial or financial relationships that could be construed as a potential conflict of interest.

## Publisher’s note

All claims expressed in this article are solely those of the authors and do not necessarily represent those of their affiliated organizations, or those of the publisher, the editors and the reviewers. Any product that may be evaluated in this article, or claim that may be made by its manufacturer, is not guaranteed or endorsed by the publisher.

## References

[1] The Global Drug Survey 2019 Findings. Global Drug Survey. (2019). Available at: https://www.globaldrugsurvey.com/gds-2019/

[2] Kaar SJ Ferris J Waldron J Devaney M Ramsey J Winstock AR. Up: the rise of nitrous oxide abuse. An international survey of contemporary nitrous oxide use. J Psychopharmacol. (2016) 30:395–401. doi: 10.1177/0269881116632375, PMID: 26912510

[3] Observatoire Français des Drogues et Toxicomanies (OFDT). Evolution de l’usage au cours de la vie de substances psychoactives (hors alcool, tabac et cannabis) parmi les 17 ans-OFDT. (2019). Available at: https://www.ofdt.fr/statistiques-et-infographie/series-statistiques/evolution-de-l-usage-au-cours-de-la-vie-de-substances-psychoactives-hors-alcool-tabac-et-cannabis-parmi-les-17-ans/

[4] Observatoire Français des Drogues et Toxicomanies (OFDT). Psychoactive substances, users and markets - recent trends (2019-2020). Tendance (2020); 141. Available at: https://en.ofdt.fr/BDD/publications/docs/eftacg2ac.pdf

[5] Drug misuse: findings from the 2018 to 2019 Crime Survey for Englang and Wales. GOV.UK. (2019). Available at: https://www.gov.uk/government/statistics/drug-misuse-findings-from-the-2018-to-2019-csew

[6] Perino J Tournier M Mathieu C Letinier L Peyré A Perret G. Psychoactive substance use among students: a cross-sectional analysis. Fundam Clin Pharmacol. (2022) 36:908–14. doi: 10.1111/fcp.12771, PMID: 35194825 PMC9544725

[7] Agence Nationale des Médicaments et Produits de Santé (ANSM). Nos missions-Organiser les vigilances-Addictovigilance. Available at: https://ansm.sante.fr/qui-sommes-nous/nos-missions/assurer-la-securite-des-produits-de-sante/p/organiser-les-vigilances#addictovigilance

[8] Code de la Santé Publique. Article R5132-113 relatif aux Centres d’évaluation et d’information sur la pharmacodépendance et d’addictovigilance. Available at: https://www.legifrance.gouv.fr/codes/article_lc/LEGIARTI000034687202/2017-05-11

[9] Code de la Santé Publique. Article R5132-114. Available at: https://www.legifrance.gouv.fr/codes/article_lc/LEGIARTI000034687195

[10] Victorri-Vigneau C Jolliet P. Scoring pharmacodependence seriousness: a novel CEIP’s evaluation tool. Therapies. (2006) 61:517–22. doi: 10.2515/therapie:2006089 17348608

[11] American Psychiatric Association. DSM-V-TM diagnostic and statiscal manual of mental disorders. 5th ed. American Psychiatric Association: Arlington, VA (2013).

[12] Association française des centres d’addictovigilance. Protoxyde d’azote. Addictovigilance (2019); 9. Available at: https://addictovigilance.fr/wp-content/uploads/spip/pdf/bulletin_addictovigilance9_site.pdf

[13] Association française des centres d’addictovigilance. Augmentation des complications sanitaires graves lors de l’usage non medical du protoxyde d’azote en France-communiqué de l’Association Française des Centres d’Addictovigilance. (2019). Available at: https://addictovigilance.fr/wp-content/uploads/spip/pdf/communique_association_addictovigilance_protoxyde_azote_5_novembre_2019.pdf

[14] Micallef J Mallaret M Lapeyre-Mestre M Daveluy A Victorri-Vigneau C Peyrière H. Warning on increased serious health complications related to non-medical use of nitrous oxide. Therapies. (2021) 76:478–9. doi: 10.1016/j.therap.2020.01.002 32063400

[15] Association française des centres d’addictovigilance. Augmentation des complications sanitaires graves associées à l’usage non médical du protoxyde d’azote en France - Communiqué. (2022). Available at: https://addictovigilance.fr/wp-content/uploads/2022/06/Communique%CC%81-protoxyde-dazote-23-juin-2022.pdf

[16] Ministère de la Santé et de la Prévention, Ministère des Solidarités, de l’Autonomie et des Personnes Handicapées. Augmentation des cas graves en lien avec l’usage détourné de protoxyde d’azote (« gaz hilarant »): les autorités sanitaires alertent sur les dangers de cette pratique-communiqué de presse. solidarité-sante.gouv.fr (2019).

[17] Ministère de la Santé et de la Prévention, Ministère des Solidarités, de l’Autonomie et des Personnes Handicapées. De nouveaux chiffres sur l’usage détourné de protoxyde d’azote (« gaz hilarant ») pour éclairer les autorités sanitaires - communiqué de presse. solidarité-sante.gouv.fr (2020). Available at: https://solidarites-sante.gouv.fr/archives/archives-presse/archives-communiques-de-presse/article/de-nouveaux-chiffres-sur-l-usage-detourne-de-protoxyde-d-azote-gaz-hilarant

[18] Agence Nationale des Médicaments et Produits de Santé (ANSM), Agence nationale de sécurité sanitaire de l’alimentation, de l’environnement et du travail (ANSES). Protoxyde d’azote: des intoxications en hausse. (2021). Available at: https://ansm.sante.fr/actualites/protoxyde-dazote-des-intoxications-en-hausse#:~:text=Un%20encadrement%20plus%20strict%20de,adopt%C3%A9e%20le%201er%20juin%202021.

[19] The Global Drug Survey 2014 Findings. Global Drug Survey. (2014). Available at: https://www.globaldrugsurvey.com/past-findings/the-global-drug-survey-2014-findings/

[20] Xiang Y Li L Ma X Li S Xue Y Yan P. Recreational nitrous oxide abuse: prevalence, neurotoxicity, and treatment. Neurotox Res. (2021) 39:975–85. doi: 10.1007/s12640-021-00352-y 33770366

[21] Huizink AC. Trends and associated risks in adolescent substance use: E-cigarette use and nitrous oxide use. Curr Opin Psychol. (2022) 45:101312. doi: 10.1016/j.copsyc.2022.101312, PMID: 35313182

[22] CEIP-Addictovigilance de Nantes, CEIP-Addictovigilance de Bordeaux, French Addictovigilance network. Rapport d’expertise, bilan d’addictovigilance, protoxyde d’azote, données 2020. (2021). Available at: https://ansm.sante.fr/uploads/2021/11/16/20211116-rapport-anonymise-protoxyde-dazote-sans-annexe-donnees-2020.pdf

[23] CEIP-Addictovigilance de Nantes, CEIP-Addictovigilance de Bordeaux, French Addictovigilance Network. Synthèse du rapport d’expertise, bilan d’addictovigilance, protoxyde d’azote, données 2018–2019. (2020). Available at: https://ansm.sante.fr/uploads/2021/01/15/20200708-rapport-addictovigilance-protoxyde-azote-2018-2019.pdf

[24] Lapeyre-Mestre M Boucher A Daveluy A Gibaja V Jouanjus E Mallaret M. Addictovigilance contribution during COVID-19 epidemic and lockdown in France. Therapies. (2020) 75:343–54. doi: 10.1016/j.therap.2020.06.006 PMC7309935 32660776

[25] Nasr SZ Nasrallah AI Abdulghani M Sweet SC. The impact of conventional and nonconventional inhalants on children and adolescents. Pediatr Pulmonol. (2018) 53:391–9. doi: 10.1002/ppul.23836 29084362

[26] Guerlais M Victorri-Vigneau C Jolliet P. Mécanismes de l’addiction: particularités des adolescents In: Cochat P, editor. Addictions chez l’enfant et l’adolescent. Montrouge: Doin (2014) 5–13.

[27] Zheng D Ba F Bi G Guo Y Gao Y Li W. The sharp rise of neurological disorders associated with recreational nitrous oxide use in China: a single-center experience and a brief review of Chinese literature. J Neurol. (2020) 267:422–9. doi: 10.1007/s00415-019-09600-w, PMID: 31655888

[28] Journal Officiel de la République Française. Loi n° 2021–695 du 1er juin 2021 tendant à prévenir les usages dangereux du protoxyde d’azote: www.legifrance.gouv.fr/jorf/id/JORFTEXT000043575111. Loi n° 2021-695 juin 2 (2021). Available at: https://www.legifrance.gouv.fr/jorf/id/JORFTEXT000043575111

[29] Maze M Fujinaga M. Pharmacology of nitrous oxide. Best Pract Res Clin Anaesthesiol. (2001) 15:339–48. doi: 10.1053/bean.2001.0166

[30] Maze M Fujinaga M. Recent advances in understanding the actions and toxicity of nitrous oxide: editorial. Anaesthesia. (2000) 55:311–4. doi: 10.1046/j.1365-2044.2000.01463.x 10781114

[31] Rosen M. Nitrous oxide for relief of labor pain: a systematic review. Am J Obstet Gynecol. (2002) 186:S110–26. doi: 10.1016/S0002-9378(02)70186-5 12011877

[32] Emmanouil DE Quock RM. Advances in understanding the actions of nitrous oxide. Anesth Prog. (2007) 54:9–18. doi: 10.2344/0003-3006(2007)54[9:AIUTAO]2.0.CO;2, PMID: 17352529 PMC1821130

[33] Sanders RD Weimann J Maze M Warner DS Warner MA. Biologic effects of nitrous oxide. Anesthesiology. (2008) 109:707–22. doi: 10.1097/ALN.0b013e3181870a17, PMID: 18813051

[34] Oussalah A Julien M Levy J Hajjar O Franczak C Stephan C. Global burden related to nitrous oxide exposure in medical and recreational settings: a systematic review and individual patient data meta-analysis. J Clin Med. (2019) 8:551. doi: 10.3390/jcm8040551 31018613 PMC6518054

[35] Zheng R Wang Q Li M Liu F Zhang Y Zhao B. Reversible neuropsychiatric disturbances caused by nitrous oxide toxicity: clinical, imaging and electrophysiological profiles of 21 patients with 6–12 months follow-up. Neuropsychiatr Dis Treat. (2020) 16:2817–25. doi: 10.2147/NDT.S270179 33262596 PMC7695601

[36] Blin J Guerlais M Masson D Catteau A Deheul S Victorri-Vigneau C. La toxicologie du protoxyde d’azote. Rev Franc Lab. (2021) 2021:48–53. doi: 10.1016/S1773-035X(21)00252-5

[37] Garakani A Jaffe RJ Savla D Welch AK Protin CA Bryson EO. Neurologic, psychiatric, and other medical manifestations of nitrous oxide abuse: a systematic review of the case literature. Am J Addict. (2016) 25:358–69. doi: 10.1111/ajad.12372, PMID: 27037733

[38] West JB. Humphry Davy, nitrous oxide, the pneumatic institution, and the royal institution. Am J Phys Lung Cell Mol Phys. (2014) 307:L661–7. doi: 10.1152/ajplung.00206.2014 25172910

[39] Ellis H. Horace Wells: pioneer of nitrous oxide anaesthesia. Br J Hosp Med. (2015) 76:56–6. doi: 10.12968/hmed.2015.76.1.56, PMID: 25585189

[40] ANSM, HAS, Assurance Maladie. Résumé des caractéristiques du produit:Kalinox 50% /50% gaz médical comprimé-Base de données publique des médicaments. (2018). Available at: https://base-donnees-publique.medicaments.gouv.fr/affichageDoc.php?specid=67577233&typedoc=R

[41] Shimizu T Nishimura Y Fujishima Y Miyajima H Honda N. Subacute myeloneuropathy after abuse of nitrous oxide: an electron microscopic study on the peripheral nerve. Rinsho Shinkeigaku. (1989) 29:1129–35. 2557181

[42] van Amsterdam J Nabben T van den Brink W. Recreational nitrous oxide use: prevalence and risks. Regul Toxicol Pharmacol. (2015) 73:790–6. doi: 10.1016/j.yrtph.2015.10.017, PMID: 26496821

[43] Dong X Ba F Wang R Zheng D. Imaging appearance of myelopathy secondary to nitrous oxide abuse: a case report and review of the literature. Int J Neurosci. (2019) 129:225–9. doi: 10.1080/00207454.2018.1526801, PMID: 30234413

[44] Sun W Liao JP Hu Y Zhang W Ma J Wang GF. Pulmonary embolism and deep vein thrombosis caused by nitrous oxide abuse: a case report. World J Clin Cases. (2019) 7:4057–62. doi: 10.12998/wjcc.v7.i23.4057, PMID: 31832409 PMC6906557

[45] Marotta DA Kesserwani H. Nitrous oxide induced posterior cord myelopathy: beware of the methyl folate trap. Cureus. (2020). Available at: https://www.cureus.com/articles/36425-nitrous-oxide-induced-posterior-cord-myelopathy-beware-of-the-methyl-folate-trap 10.7759/cureus.9319 PMC7444745 32850197

[46] Selvaraj A Wong KE. An unusual case of ‘laughing gas’ addiction in Singapore. Eur Psychiatry. (2017) 41:s878–8. doi: 10.1016/j.eurpsy.2017.01.1772

[47] Berger-Vergiat A Pellereau K Boucher A. Severe nitrous oxide use disorder: a case-report. Toxicol Anal Clin. (2019) 31:S78. doi: 10.1016/j.toxac.2019.03.124

[48] Mancke F Kaklauskaitė G Kollmer J Weiler M. Psychiatric comorbidities in a young man with subacute myelopathy induced by abusive nitrous oxide consumption: a case report. Subst Abuse Rehabil. (2016) 7:155–9. doi: 10.2147/SAR.S114404, PMID: 27729826 PMC5047713

[49] Fidalgo M Prudhomme T Allio A Bronnec M Bulteau S Jolliet P. Nitrous oxide: what do we know about its use disorder potential? Results of the French monitoring Centre for Addiction network survey and literature review. Subst Abus. (2019) 40:33–42. doi: 10.1080/08897077.2019.1573210, PMID: 30913001

[50] Gillman MA. Nitrous oxide abuse in perspective. Clin Neuropharmacol. (1992) 15:297–306. PMID: 1516075 10.1097/00002826-199208000-00004

[51] Gillman MA. Nitrous oxide has a very low abuse potential. Addiction. (1995) 90:439–40. doi: 10.1111/j.1360-0443.1995.tb03791.x 7794392

[52] Caldera A Mora J Kotler M Eiger G. Pulmonary embolism in a patient with pernicious anemia and Hyperhomocysteinemia. Chest. (2002) 122:1487–8. doi: 10.1378/chest.122.4.1487, PMID: 12377886

[53] Martinelli I Battaglioli T Pedotti P Cattaneo M Mannucci PM. Hyperhomocysteinemia in cerebral vein thrombosis. Blood. (2003) 102:1363–6. doi: 10.1182/blood-2003-02-0443 12714502

[54] Cantu C Alonso E Jara A Martínez L Ríos C de Fernández M. Hyperhomocysteinemia, low folate and vitamin B _12_ concentrations, and methylene tetrahydrofolate reductase mutation in cerebral venous thrombosis. Stroke. (2004) 35:1790–4. doi: 10.1161/01.STR.0000132570.24618.78, PMID: 15192249

[55] Oomens T Fokkema TMM van den Bogaard B de Metz J van Nieuwenhuizen RC Riezebos RK. Thromboembolisms due to recreational use of nitrous oxide. Ned Tijdschr Geneeskd. (2021) 165:D5607. 33914433

[56] den Uil SH Vermeulen EGJ Metz R Rijbroek A de Vries M. Aortic arch thrombus caused by nitrous oxide abuse. J Vasc Surg Cases Innov Tech. (2018) 4:80–2. doi: 10.1016/j.jvscit.2018.01.001 29942888 PMC6013293

[57] Agarwal P Khor SY Do S Charles L Tikaria R. Recreational nitrous oxide-induced subacute combined degeneration of the spinal cord. Cureus. (2021) 13:e19377. doi: 10.7759/cureus.19377, PMID: 34909324 PMC8653952

[58] Norris F Mallia P. Lesson of the month 2: a case of nitrous oxide-induced pancytopenia. Clin Med. (2019) 19:129–30. doi: 10.7861/clinmedicine.19-2-129, PMID: 30872294 PMC6454366

[59] Glijn NHP van der Linde D Ertekin E van Burg PLM Grimbergen YAM Libourel EJ. Is nitrous oxide really that joyful? Neth J Med. (2017) 75:304–6. PMID: 28956785

[60] Paulus MC Wijnhoven AM Maessen GC Blankensteijn SR van der Heyden MAG. Does vitamin B12 deficiency explain psychiatric symptoms in recreational nitrous oxide users? A narrative review. Clin Toxicol. (2021) 59:947–55. doi: 10.1080/15563650.2021.1938107, PMID: 34348072

[61] Chien WH Huang MC Chen LY. Psychiatric and other medical manifestations of nitrous oxide abuse: implications from case series. J Clin Psychopharmacol. (2020) 40:80–3. doi: 10.1097/JCP.0000000000001151, PMID: 31809285

[62] Cousaert C Heylens G Audenaert K. Laughing gas abuse is no joke. An overview of the implications for psychiatric practice. Clin Neurol Neurosurg. (2013) 115:859–62. doi: 10.1016/j.clineuro.2013.04.004, PMID: 23643142

[63] Lan SY Kuo CY Chou CC Kong SS Hung PC Tsai HY. Recreational nitrous oxide abuse related subacute combined degeneration of the spinal cord in adolescents - a case series and literature review. Brain Dev. (2019) 41:428–35. doi: 10.1016/j.braindev.2018.12.003, PMID: 30611595

[64] ANSM, HAS, l’Assurance Maladie. Base de données publique des médicaments. Available at: http://base-donnees-publique.medicaments.gouv.fr/index.php

[65] IBM. IBM Micromedex. Available at: https://www.micromedexsolutions.com/

