# Erratum: Nitrous oxide: a unique official French addictovigilance national survey

**DOI:** 10.3389/fpubh.2023.1235720

**Published:** 2023-07-04

**Authors:** Frontiers Production Office

**Affiliations:** Frontiers Media SA, Lausanne, Switzerland

**Keywords:** nitrous oxide, substance use disorder, addictovigilance, pharmacodependence, addiction

Due to a production error, the consortium French Addictovigilance Network was omitted from the authors list.

There has also been an acknowledgments section added that lists the following names, crediting their involvement with the consortium.

Alexandra Boucher, Anne Batisse, Anne Roussin, Bernard Fauconneau, Cécile Chevallier, Frédéric Libert, Hélène Peyrière, Joëlle Micallef, Juliana Tournebize, Maryse Lapeyre-Mestre, Michel Spadari, Nathalie Fouilhé, Reynald Le Boisselier, Sylvie Deheul, Valérie Gibaja.

The publisher apologizes for this mistake. The original article has been updated.

